# Giant fibroepithelial polyp of the sigmoid colon

**DOI:** 10.1016/j.igie.2023.09.008

**Published:** 2023-09-28

**Authors:** Talia F. Malik, Neil R. Sharma

**Affiliations:** 1 Department of Internal Medicine, Chicago Medical School at Rosalind Franklin University of Medicine and Science, North Chicago, Illinois, USA; 2 Department of Gastroenterology, Parkview Cancer Institute, Fort Wayne, Indiana, USA

A 65-year-old woman with a history of hypertension, GERD, diabetes, and hyperlipidemia was referred for evaluation of an asymptomatic mass found in the sigmoid colon during a routine screening colonoscopy.

The endoscopic evaluation showed a 5- to 6-cm smooth polypoid mass with a long stalk attached to the mucosal surface of the sigmoid colon. The mucosal surface was evaluated by high-definition white-light and narrow-band imaging (Fig. 1A). The mass was resected via endoscopic submucosal dissection using a pocket technique with a partial circumferential incision followed by submucosal dissection. An Olympus Jr. 2.3 SB knife (Olympus America, Center Valley, Penn, USA) was used for en bloc resection (Fig. 1B and 1C). The lesion was injected with saline, 1:200,000 epinephrine, methylene blue, and hetastarch. An initial incision was made, and an upper endoscope fitted with a distal cap was used to enter the submucosal space. Dissection was then performed distal to proximally. Total resection time was 21 minutes. The gross examination of the resected specimen showed a 6.5 × 2.8 × 2.5 cm pedunculated mass tan to pink in color (Fig. 1D and 1E). Microscopic examination showed expansive proliferation of benign fibrous, vascular and adipose tissue underlying normal colonic mucosa consistent with giant fibroepithelial polyp (Fig. 1F).

**Figure 1:**
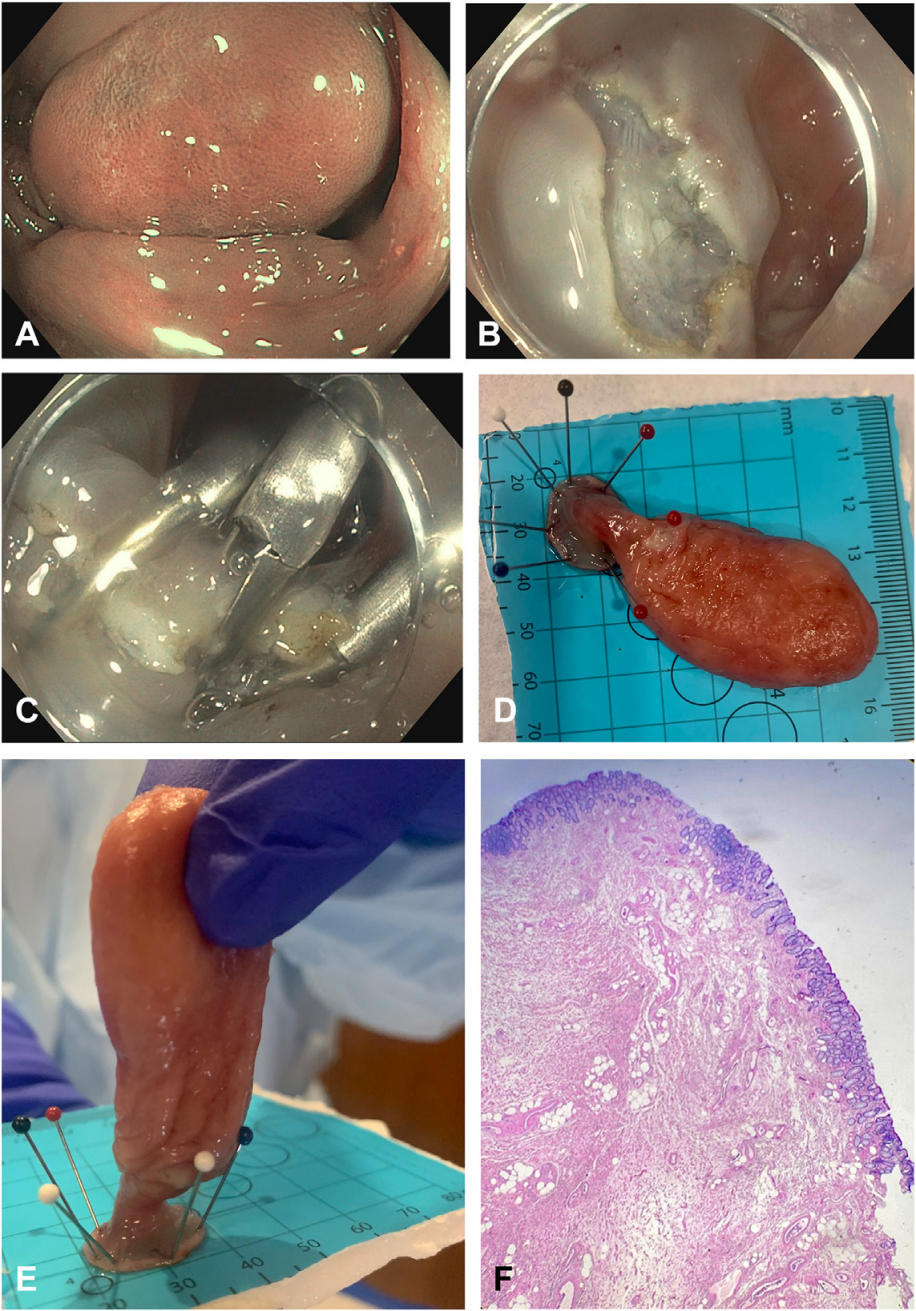
**A,** Endoscopic appearance of a giant fibroepithelial polyp in the sigmoid colon. **B,** Endoscopic appearance of the resection site after giant fibroepithelial polyp resection using endoscopic submucosal dissection. **C,** Endoscopic appearance after giant fibroepithelial polyp resection showing prophylactic clip placement. **D,** Gross examination of the resected specimen showing 6.5 × 2.8 × 2.5 cm pedunculated mass tan to pink in color (*plan view*). **E,** Gross examination of the resected specimen showing 6.5 × 2.8 × 2.5 cm pedunculated mass tan to pink in color (*profile view*). **F**, Microscopic examination showing expansive proliferation of benign fibrous, vascular and adipose tissue underlying normal colonic mucosa (H&E, orig. mag. x40).

Fibroepithelial polyps are benign tumors of mesodermal origin that are uncommon in the GI tract, with the majority found in the esophagus. Although benign, fibroepithelial polyps >3 cm originating in the sigmoid colon are rare and should be differentiated from malignant tumors by endoscopic and histologic evaluation.^1^, ^2^, ^3^ Giant fibroepithelial polyps have been associated with intestinal obstruction, and timely resection may prevent this adverse event.^4^^,^^5^ This case shows the feasibility of endoscopic submucosal dissection for resection of a giant fibroepithelial polyp in an unusual location.

Informed consent was obtained for this case report.

## Disclosure

N. R. Sharma: Consultant for Boston Scientific, Medtronic, STERIS, and Olympus. T. F. Malik: No financial relationships.
